# Enhanced therapeutic effects of mesenchymal stem cell-derived extracellular vesicles within chitosan hydrogel in the treatment of diabetic foot ulcers

**DOI:** 10.1007/s10856-023-06746-y

**Published:** 2023-08-28

**Authors:** Shuangshuang Yang, Siyu Chen, Chengpeng Zhang, Jing Han, Chunyuan Lin, Xiaohui Zhao, Huizhen Guo, Yi Tan

**Affiliations:** Qilu Cell Therapy Technology Co., Ltd, No.1758 Gangyuan Six Road, Ji’nan, Shandong China

## Abstract

Extracellular vesicles (EVs) derived from human umbilical cord mesenchymal stem cells (hUCMSCs) have emerged as promising candidates for cell-free therapy in various diseases, including chronic cutaneous wounds. However, the lack of standardized protocols for EVs’ preparation and identification poses a significant challenge to their clinical application. Thus, the objective was to develop a safe and efficient method for the large-scale production of hUCMSC-derived EVs while establishing a comprehensive identification protocol encompassing morphology, particle size distribution, protein expression, and purity. This study observed that most of the EVs acquired through the protocol exhibited either a cup-shaped or round-shaped structure, with a median diameter of ~73.25 nm. The proportions of EVs positive for CD9, CD63, and CD81 were 37.5%, 38.6%, and 19.8%, respectively. To enhance their therapeutic potential in wound treatment, EVs were incorporated into chitosan hydrogel, forming chitosan hydrogel-EVs (CS-EVs). Furthermore, it was demonstrated that CS-EVs exhibited continuous release of EVs into the surrounding environment and, importantly, that the released EVs were internalized by human umbilical vein endothelial cells (HUVECs), resulting in significant enhancement of cell migration and angiogenesis. Additionally, in a rat model of diabetic foot ulcers, CS-EVs demonstrated a robust therapeutic effect in promoting wound healing. Following a 15-day treatment period, the group treated with CS-EVs demonstrated an impressive 93.3% wound closure ability, accompanied by a high degree of re-epithelialization. In contrast, the control group exhibited only a 71.5% reduction in wound size. In summary, this study offers solutions for the purification, characterization, and application of EVs in clinical wound treatment. These results not only offer fresh perspectives on the involvement of hUCMSC-derived EVs in wound healing but also introduce a non-invasive approach for applying EVs that holds practical significance in skin repair.

**Graphical Abstract:**

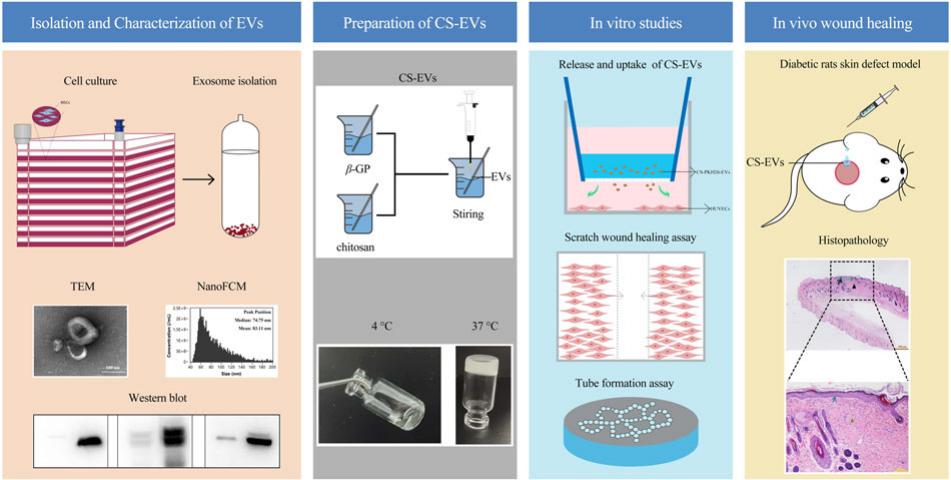

## Introduction

Diabetes mellitus is a chronic metabolic disease characterized by persistent high blood sugar levels and is caused by multiple factors. Due to its high rates of mortality and morbidity, diabetes has become a significant global health issue. Recent years have seen a rise in diabetes incidence due to an aging population and changes in dietary patterns [1, 2]. Diabetes can lead to various complications affecting organs such as the heart, brain, kidneys, peripheral nerves, eyes, and feet. Diabetic foot ulcer (DFU) is a severe and complicated complication associated with diabetes [3, 4]. The development of DFU is attributed to diabetic neuropathy, peripheral vascular disease, and ulcer formation. Various factors, including infection, hyperglycemia, and impaired angiogenesis, play significant roles in causing delayed or non-healing ulcer wounds [5, 6]. Current treatment approaches for DFU mainly focus on blood sugar control, local debridement, and wound care. Unfortunately, there is still a lack of effective solutions to promote the healing of complex wounds [7].

Recent studies have demonstrated that mesenchymal stem cells (MSCs) play a role in angiogenesis, epithelial regeneration, granulation formation, and closure of chronic wounds by secreting cytokines, chemokines, growth factors, and other factors [8–11]. MSC-based therapy for DFU has shown promising regenerative potential [12]. Nevertheless, MSCs have a restricted lifespan within wound tissue and typically vanish within 24 h, which presents a challenge for their practical use in wound treatment [13]. Moreover, concerns regarding potential tumorigenicity and undesirable immune responses associated with MSC transplantation remain unresolved in the context of DFU therapy [14].

Extracellular vesicles (EVs) released by living cells are crucial for intercellular communication. These vesicles transport bioactive molecules, including proteins, mRNAs, and microRNAs, to recipient cells, exerting significant effects on cellular behavior [15]. EVs exhibit similar beneficial functions to their parent cells [16, 17]. MSC-derived EVs have been identified as functional mediators of MSCs, exhibiting immune regulatory properties and promoting tissue regeneration [18, 19]. Importantly, MSC-derived EVs display a lower potential for triggering immune responses, enhancing their safety profile [20]. Given their clinical safety profile, EVs may present distinct advantages over stem cell therapy. Nevertheless, the intricate nature of tissue repair and the immune system’s prompt clearance of free EVs impose limitations on their therapeutic effectiveness [21]. Thus, a challenge in utilizing EVs for wound treatment is to mitigate their rapid clearance. Chitosan hydrogel, a thermosensitive material widely used as a carrier in various treatments, holds promise in this regard. Through cross-linking, cells or drugs can be incorporated into the chitosan hydrogel, a biocompatible polymer [22], enabling slow release of the encapsulated substances and prolonging their in situ retention time [23]. Therefore, incorporating EVs into chitosan hydrogel can attenuate their rapid clearance, enhancing their beneficial effects on wound tissue recovery.

In the last few years, advanced therapies for wound healing and tissue repair have included the utilization of EVs and biomaterials [24]. For example, researchers have suggested an innovative and advantageous approach for wound healing and dermal tissue replacement by employing EVs derived from human endometrial stem cells encapsulated in chitosan hydrogel [25]. Similarly, in a diabetic rat skin defect model, the combination of EVs derived from gingival mesenchymal stem cells and chitosan/silk hydrogel sponge has demonstrated accelerated wound healing [7]. Furthermore, a highly efficient injectable self-healing methylcellulose-chitosan hydrogel loaded with exosomes from human placental cell culture, linked by Schiff base, has been developed for the treatment of severe wounds [26]. Additionally, emerging evidence suggests that EVs derived from human adipose-derived mesenchymal stem cells, when incorporated into a self-healing injectable hydrogel, promote diabetic wound healing by regulating macrophage polarization and accelerating angiogenesis [27]. These studies demonstrate that combining EVs with biomaterials reduces EVs clearance, allowing them to remain at the treatment site and thereby enhancing treatment efficacy. Although EV-based biomaterials have shown promising results in wound healing, there is a limited number of clinical trials conducted on them. Therefore, before the large-scale application of MSC-EVs in humans, various clinical challenges and potential limitations need to be addressed [18, 28]. Standardized preparation procedures and identification protocols for EVs, as well as clarification of the proportions of each EV subpopulation based on different isolation methods, are critical factors to ensure consistent outcomes. Additionally, the source of EVs is an important consideration in clinical applications, as it can influence the outcome of wound healing.

Among various sources, EVs derived from human umbilical cord mesenchymal stem cells (hUCMSCs) offer economic advantages and are subject to regulatory compliance. This study developed an efficient protocol for isolating and characterizing hUCMSC-derived EVs. Subsequently, these EVs were integrated with chitosan hydrogel, resulting in the formation of CS-EVs, showcasing improved efficacy in wound treatment. Moreover, further evaluations were conducted to examine the favorable impacts of CS-EVs on endothelial cells and a rat model of diabetic foot ulcers. These findings suggest that incorporating EVs into chitosan hydrogel represents a novel approach to maintaining the therapeutic efficacy of hUCMSC-derived EVs in promoting efficient skin healing.

## Materials and methods

### Cell culture

The hUCMSCs (Passages 3–6), provided by Shandong Cell-Tissue Bank, were cultured and expanded using Dulbecco’s Modified Eagle Medium F-12 (DMEM/F12) supplemented with 10% bovine fetal bovine serum (FBS) and 100 U/mL penicillin-streptomycin. Human umbilical vein endothelial cells (HUVECs) were procured from ATCC (Manassas, VA) and cultivated in Dulbecco’s Modified Eagle Medium (DMEM) supplemented with 10% FBS and 100 U/mL penicillin-streptomycin. The basal medium, FBS, and PBS used in cell culture experiments were acquired from Gibco (Grand Island, USA) and were subjected to ultracentrifugation at 160,000 *g* for 8 h to eliminate vesicle analogs prior to use.

### Characterization of hUCMSCs

Flow cytometry analysis was performed to assess the surface antigen expression of hUCMSCs. A 100 µL suspension containing hUCMSC cells (5 × 10^7^ cells/mL) was incubated with 5 µL of PE-conjugated antibodies specific to CD90, CD105, CD34, and CD45, respectively, for 20 min at room temperature. After centrifugation at 1000 *g* for 5 min, the pelleted cells were collected, washed twice with PBS, and finally resuspended in 200 µL of PBS for flow cytometry analysis using a BD Biosciences instrument. All antibodies used in the experiment were obtained from BD Biosciences.

### Multidirectional identification of umbilical cord-MSCs (hUCMSCs)

Passage 6 of hUCMSCs was seeded in six-well plates and cultured using a suitable differentiation medium as per the manufacturer’s instructions [29]. To induce osteogenic differentiation, each well of the six-well plate was coated with 1 mL of 0.1% gelatin solution at room temperature for 30 min to prevent the floating of hUCMSCs during induction. A 4 mL suspension of hUCMSC cells (1 × 10^4^ cells/mL) was then seeded in the six-well plates and maintained in DMEM/F12 supplemented with 10% FBS and 100 U/mL penicillin-streptomycin. The following day, the original medium was replaced with induction medium containing osteogenic differentiation supplements. Osteogenically differentiated cells were stained with alizarin red on day 21. The cell density was set to 1 × 10^4^ cells/mL using a complete medium, and 4 mL of the cell suspension was plated onto six-well plates and incubated for 24 h. Following this, a medium containing adipogenic differentiation supplements was applied for a 14-day adipogenic differentiation period. After differentiation, the cells were stained with oil red O. For chondrogenic differentiation, the hUCMSCs were suspended in a medium containing a chondrogenic differentiation supplement. Approximately 2 mL of cells (2 × 10^5^ cells/mL) were seeded in a 15 mL centrifuge tube and centrifuged at 150 g for 5 min to allow the cells to settle at the bottom of the tube. The cells were maintained at 37 °C for 24 h, and then the tube was gently shaken to suspend the cell precipitate clumps. On day 21, the cells that underwent chondrogenic differentiation were subjected to staining with Alcian blue and safranin O. All the necessary reagents were sourced from StemCell Technologies.

### Enrichment of hUCMSC-derived extracellular vesicles

To isolate EVs, a substantial amount of culture medium was required. This medium was collected by seeding hUCMSCs at a density of 8000 cells/cm^2^ in the Cell Factory System. When the cells reached 80–90% confluency, the existing medium was collected [30] and stored at –80 °C. The collected medium from multiple batches was pooled together, resulting in a total volume of 6 L. Subsequently, the collected medium underwent centrifugation at 16,000 *g* for 30 min at 4 °C to remove whole cells and excessive cellular debris. To enrich the EVs in the supernatant, the protocol described by Rider et al. [31] was followed with slight modifications, including the addition of PEG 6000 to achieve a final concentration of 8%. The samples were mixed thoroughly and incubated for 12 h at 4 °C. Following incubation, the samples underwent centrifugation at 12,000 *g* for 1 h at 4 °C. The resulting pellet was reconstituted in 10 mL of PBS, and then subjected to ultracentrifugation at 120,000 *g* for 60 min to isolate EVs. The EV solutions were subsequently stored at –80 °C for further processing.

### Transmission electron microscopy (TEM)

Transmission electron microscopy (TEM) was employed to examine the morphologies of EVs. EV samples were deposited onto a carbon grid and allowed to adsorb for 20 min. Subsequently, the EVs were fixed in 1% glutaraldehyde for 5 min, washed with PBS, and visualized using a transmission electron microscope (Hitachi, Japan) [32].

### Western blot analysis

The expression of exosomal markers in the isolated EVs was analyzed using western blotting. Approximately 10 µg of protein from both EVs and hUCMSC samples were loaded onto a 12% SDS-PAGE gel (Beyotime, China) and transferred to PVDF membranes (Beyotime, China). The blots were obstructed using a blocking solution (5% milk and 0.05% Tween-20 in PBS) at room temperature for 2 h, followed by overnight incubation at 4 °C with primary antibodies against CD9, CD63, CD81, and GM130 monoclonal antibodies (2 µL antibody and 0.05% Tween-20 in 10 mL PBS, Proteintech, USA). Subsequently, secondary horseradish peroxidase-conjugated antibodies (2 µL antibody and 0.05% tween-20 in 10 mL PBS, Proteintech, USA) were used to visualize the bands using a chemiluminescent detection system (Bio-Rad, USA) and an enhanced chemiluminescence kit (Thermo Scientific, USA).

### High-sensitivity flow cytometry analysis

In order to accurately measure the particle concentration in high-sensitivity flow cytometry analysis (Flow NanoAnalyzer U30, NanoFCM), the EV suspension was diluted with filtered PBS at a ratio of 1:1000. For immunofluorescence staining analysis, 2.5 µL of PE-conjugated CD9, CD63, or CD81 antibodies were added to a 25 µL EV suspension. To assess the co-expression of CD9, CD63, and CD81 in individual vesicles, double immunofluorescence staining analysis was performed using PE-conjugated and PerCP Cy5.5-conjugated antibodies. All antibodies were sourced from BD Biosciences. To assess purity, 5 µL of 10% Triton X-100 (Sigma-Aldrich, USA) was added to a 45 µL EV suspension. The effects were evaluated by comparing the representative SSC burst traces of EVs before and after Triton X-100 treatment using the NanoFCM assay [33].

### Preparation and characterization of chitosan hydrogel

The preparation of chitosan hydrogel followed a previously reported method [34]. Chitosan powder (Hidebei, China) was dissolved in 0.1 M acetic acid to create a stock solution of 2% chitosan. Likewise, a 50% solution of *β*-glycerophosphate (*β*-GP, Yuanye, China) was prepared in sterile water, and both solutions were subsequently filtered and stored at 4 °C for future use. For the experiment, the 50% *β*-GP and 2% chitosan solutions were combined in a water bath at a volume ratio of 5:1 and stirred at 37 °C to generate a chitosan hydrogel. Subsequently, the EVs (1 × 10^11^ particles) were thoroughly mixed with five volumes of chitosan hydrogel by stirring in an ice bath to obtain the chitosan hydrogel-incorporated EVs (CS-EVs). Finally, the rheological properties of the chitosan hydrogel and CS-EVs at different temperatures (ranging from 4 to 37 °C) were investigated by measuring the elastic moduli (G’) and viscous moduli (G”) using a parallel plate rheometer.

### In vitro study of the release dynamics of CS-EVs

The in vitro release of EVs from the chitosan hydrogel was assessed using the Transwell method. Firstly, the chitosan hydrogel was mixed with EVs (1 × 10^11^ particles) to create CS-EVs, as described earlier. The CS-EVs solution was then loaded onto the upper chamber of the Transwell, ~200 µL per well, and placed in a 24-well plate. Gelatinization was performed for 10 min at 37 °C to form the CS-EVs gel. Next, the CS-EVs gel was submerged in 1.5 mL of PBS at 37 °C for different time intervals, spanning up to 3 days. At each time point, supernatants were collected, and the release ratio of EVs was assessed using HSFCM analysis.

### In vitro study of the uptake dynamics of CS-EVs

The EVs were labeled with PKH26 (Sigma-Aldrich, USA) following the manufacturer’s instructions with slight modifications. The EVs pellets were first resuspended in 2 mL of dilution C and gently mixed. Subsequently, the EVs solutions were incubated with 10 µM PKH26 for 5 min at room temperature. The staining process was halted by adding an equal volume of 1% BSA, after which the mixture was centrifuged at 120,000 *g* for 60 min to eliminate free PKH26. The PKH26-labeled exosomes were then resuspended in 1 mL of PBS for further experiments. To investigate the uptake behavior of EVs released from CS-EVs in HUVEC cells, the same method as described above was employed. The preparation of CS-EVs (1 × 10^11^ particles) was similar to the aforementioned procedure, where the CS-EV solution was loaded onto the upper chamber of the Transwell. Subsequently, HUVEC cells were seeded in a 24-well plate at a density of 6000 cells/cm^2^. Following a 12 h incubation, the HUVECs were gently washed with PBS and fixed in 4% paraformaldehyde for 15 min. Subsequently, the cells were washed again and stained with FITC Phalloidin and DAPI (Solarbio, China). The signals emitted by the stained cells were captured using a laser scanning confocal microscopy LSM780 (Zeiss, GRE).

### Migration tests

To assess the potential effects of CS-EVs on cell migration promotion, a scratch wound healing assay was conducted. HUVECs were plated in a 24-well plate using a regular culture medium supplemented with 10% FBS. Once the cells reached 90% confluency, a scratch was made in the cell monolayer using a pipette tip. Following this, FBS-free medium containing chitosan hydrogel or CS-EVs (1 × 10^11^ particles) was introduced into the upper chamber of the Transwell, and the cells were cultured at 37 °C for an additional 22 h. Images of the scratch were captured at 0, 6, and 22 h and analyzed using ImageJ software. The negative control group consisted of untreated cells, while the positive control group consisted of extracellular vesicles.

### Tube formation assay

To evaluate the proangiogenic potential of leached EVs, an in vitro tube formation assay was conducted. Matrigel obtained from BD Biosciences was thawed at 4 °C and added to a 24-well plate at approximately 300 µL per well. HUVECs were then seeded on the Matrigel-coated plate and treated with a serum-free medium. Subsequently, the chitosan hydrogel and CS-EVs (1 × 10^11^ particles) were loaded onto the upper chamber of the Transwell and incubated for 8 h. Subsequently, images were captured using a microscope equipped with a camera system.

### Quantitative real-time PCR (qPCR)

HUVECs were seeded in a 24-well plate at a density of 6000 cells/cm^2^. Subsequently, the upper chamber of the Transwell was loaded with chitosan hydrogel and CS-EVs (1 × 10^11^ particles), and the cells were cultured at 37 °C for an additional 48 h. The expression levels of VEGF, bFGF, ANG-1, and GAPDH genes in different groups were analyzed using quantitative real-time PCR (qPCR) [34]. Primers for qPCR amplification (listed in Table 1) were designed using the primer 5.0 software. The qPCR assay was conducted in triplicate and repeated three times. The GAPDH gene was chosen as the reference gene to standardize the experimental data. The relative mRNA levels of VEGF, bFGF, and ANG-1 were calculated using the comparative Ct method (2^–ΔΔCT^) based on the Ct values obtained for the target genes and GAPDH. The ABI StepOnePlus PCR system (Applied Biosystems) was used for qPCR data analysis.

**Table 1:** Sequences of the primers for qPCR used in present study

Primer	Sequence (5′-3′)	Accession Number	Function
P1(S)	TGTCTAATGCCCTGGAGCCT	NM_001025366.3	VEGE
P2(AS)	GTCACATCTGCAAGTACGTTCG		
P3(S)	AGAAGAGCGACCCTCACATCA	NM_002006.6	bFGF
P4(AS)	CGGTTAGCACACACTCCTTTG		
P5(S)	AGCGCCGAAGTCCAGAAAAC	XM_047421699.1	ANG-1
P6(AS)	TACTCTCACGACAGTTGCCAT		
P7(S)	AGGGCTGCTTTTAACTCTGGT	NM_001256799.3	GAPDH
P8(AS)	CCCCACTTGATTTTGGAGGGA		

S and AS represent sense and antisense primers, respectively

### In vivo animal experiments

Sprague-Dawley (SD) rats, weighing 240 ± 20 g, were obtained from SPF Biotechnology Co., Ltd. The animal procedures were approved by the Medical Ethics Committee of Qilu Hospital of Shandong University, and all animal studies followed the International Guiding Principles for Biomedical Research Involving Animals. Diabetes was induced in the rats by administering a high-sucrose and high-fat diet for 10 weeks, followed by intraperitoneal injections of STZ (35 mg/kg, Sigma) during the 10th and 11th weeks. The confirmation of successful establishment of the diabetic rat model was based on fasting blood glucose levels consistently exceeding 11 mmol/L for more than 4 weeks.

Subsequently, 24 rats were anesthetized and randomly divided into four groups of 6 animals each. The dorsal hairs of the rats were shaved, and a full-thickness wound with a diameter of 10 mm was created. The four groups were assigned to evaluate the effects of different treatments on wound healing: hydrogel without EVs, CS-EVs (1 × 10^10^ particles), EVs (1 × 10^10^ particles), or PBS. The wounds were then covered with gauze and 3 M Tegaderm film to prevent infection. After 5, 10, or 15 days post-surgery, the wound area was photographed, and the dressing was changed in each group accordingly. Finally, after 15 days post-surgery, all rats were sacrificed, and the wound skin samples were collected for hematoxylin and eosin (H&E) staining.

### Statistical analysis

All experiments were conducted in triplicate and repeated at least three times. Statistical analysis was performed using SPSS 18.0 software. The data were presented as mean ± SD, and significance was defined as *P* < 0.05.

## Results

### Phenotypes of hUCMSCs and multidirectional identification

**Fig. 1:**
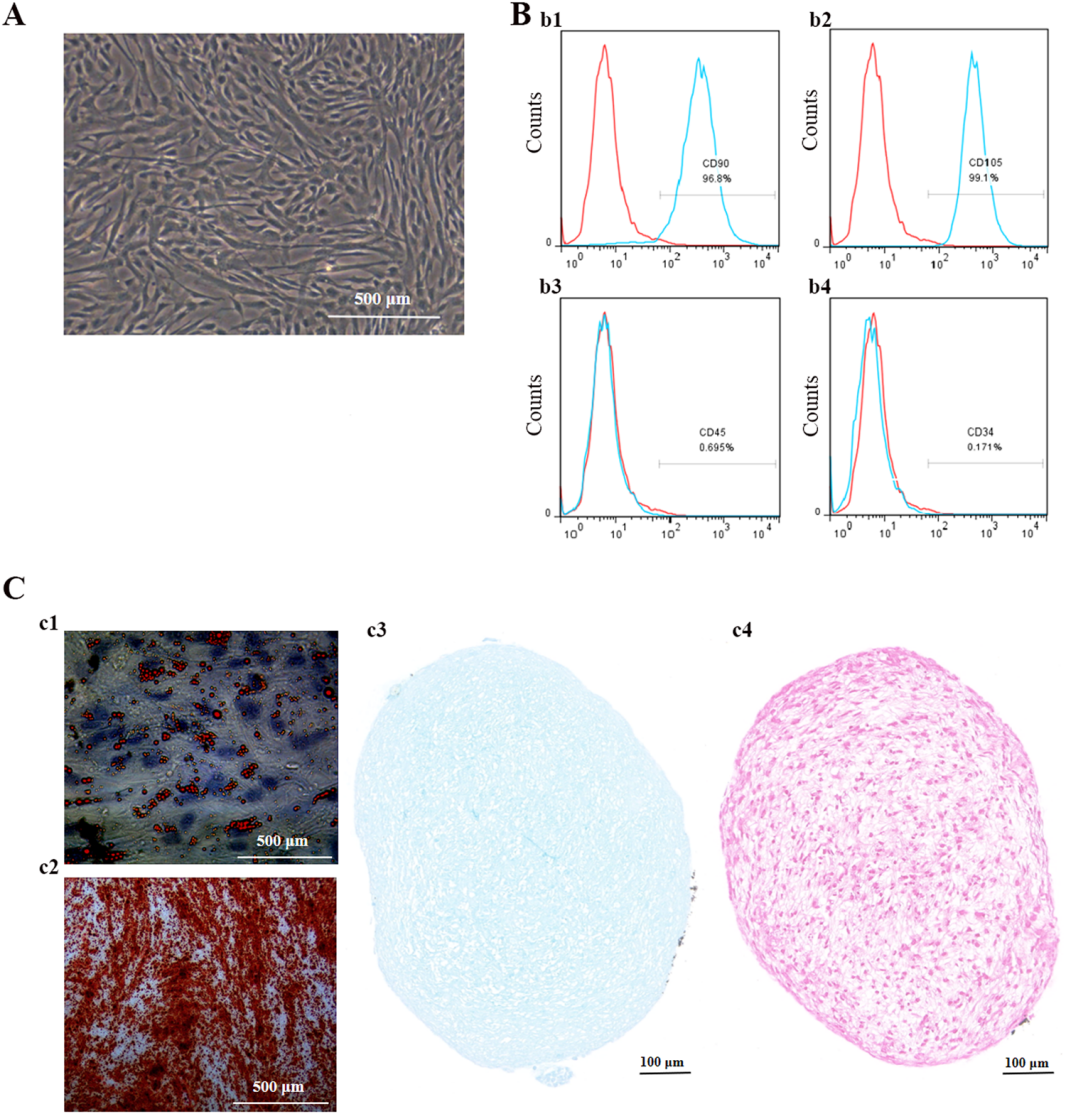
Characterization of hUCMSCs: **A** Morphology of hUCMSCs. **B** Surface markers of hUCMSCs. The cells were positive for CD90 (b1) and CD105 (b2), and negative for CD45 (b3) and CD34 (b4). **C** Multidirectional identification of hUCMSCs: c1. Oil red staining of hUCMSCs. c2. Alizarin red staining of hUCMSCs. c3. Alcian blue staining of hUCMSCs. c4. Safranin O staining of hUCMSCs

The hUCMSCs exhibited the characteristic spindle-like morphology, as depicted in Fig. 1A. Immunostaining analysis demonstrated positive expression of CD90 (96.8%) and CD105 (99.1%), while negative expression of CD45 (0.695%) and CD34 (0.171%), as depicted in Fig. 1B. CD90 and CD105 are well-established markers for hUCMSCs, whereas CD34 and CD45 are commonly associated with hematopoietic stem cells. Additionally, hUCMSCs exhibited differentiation potential towards adipogenic, osteogenic, and chondrogenic lineages, as observed in Fig. 1C. Thus, these findings affirm the suitability of hUCMSCs for culturing and harvesting conditioned medium to isolate EVs.

### Isolation and characterization of EVs

**Fig. 2:**
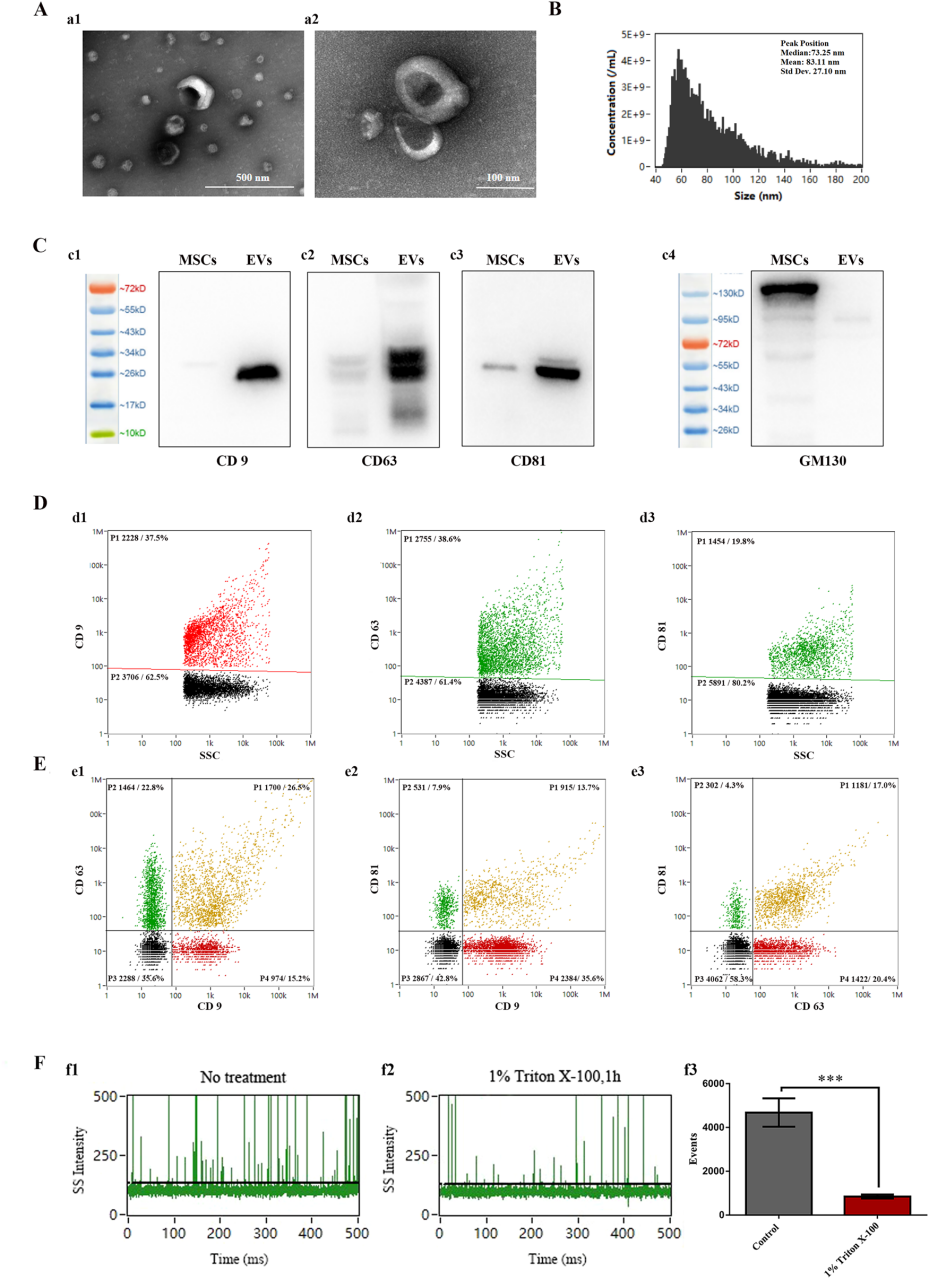
Characterization of EVs derived from hUCMSCs: **A** TEM images of EVs; Scale bar: 500 nm (a1), Scale bar: 100 nm (a2). **B** Particle size distribution analysis of EVs. **C** Expression of CD9 (c1), CD63 (c2), CD81 (c3), and GM130 (c4) markers in hUCMSCs and EVs. **D** HSFCM analysis of CD9 (d1), CD63 (d2), and CD81 (d3) in EVs derived from hUCMSCs. **E** Co-expression analysis of CD9, CD63, and CD81 in EVs. Subpopulations of EVs co-expressing CD9/CD63 (e1), CD9/CD81 (e2), and CD63/CD81 (e3). **F** Representative SSC burst traces of EVs before (f1) and after (f2) 1% Triton X-100 treatment for 1 h. (f3) Bar graph representing the event rate detected in 1 min for two samples

In order to enrich EVs, we employed a combination of PEG and ultracentrifugation techniques. Furthermore, we employed multiple methods to evaluate the concentration, quality, morphology, and purity of the isolated EVs. The majority of EVs obtained using our protocol exhibited either a cup-shaped or round-shaped morphology, as illustrated in Fig. 2A. To assess the particle concentration and size distribution of EVs isolated from hUCMSC supernatants using our protocol, we performed HSFCM analysis. NanoFCM assay results revealed an average concentration of EVs at approximately (3.97 ± 0.044) × 10^11^ particles/mL. Concurrently, the nanoFCM data for hUCMSC-derived EVs indicated a median particle size of 73.25 nm and a mean size of 83.11 nm (Fig. 2B). Western blot analysis verified the presence of exosomal markers CD9, CD63, and CD81 in the EVs isolated from hUCMSC supernatants. In contrast, the expression of GM130, which was highly expressed in parental cells, was scarcely detectable in EVs (Fig. 2C). Subpopulation analysis of EVs was conducted using the fluorescence mode of nanoFCM, revealing that the percentages of CD9-positive, CD63-positive, and CD81-positive EVs were 37.5, 38.6, and 19.8%, respectively (Fig. 2D). Similarly, the subpopulations of EVs co-expressing CD9/CD63, CD9/CD81, and CD63/CD81 were observed at 26.5%, 13.7%, and 17%, respectively (Fig. 2E). Figure 2F(f1) and (f2) present representative SSC burst traces of EVs before and after treatment with 1% Triton X-100 for 1 h. A significant reduction in event rate was observed for EVs following detergent treatment. EVs isolated from hUCMSC cell culture supernatants contained 81.80 ± 1.65% of membrane vesicles.

### Characterization of the chitosan hydrogel

**Fig. 3:**
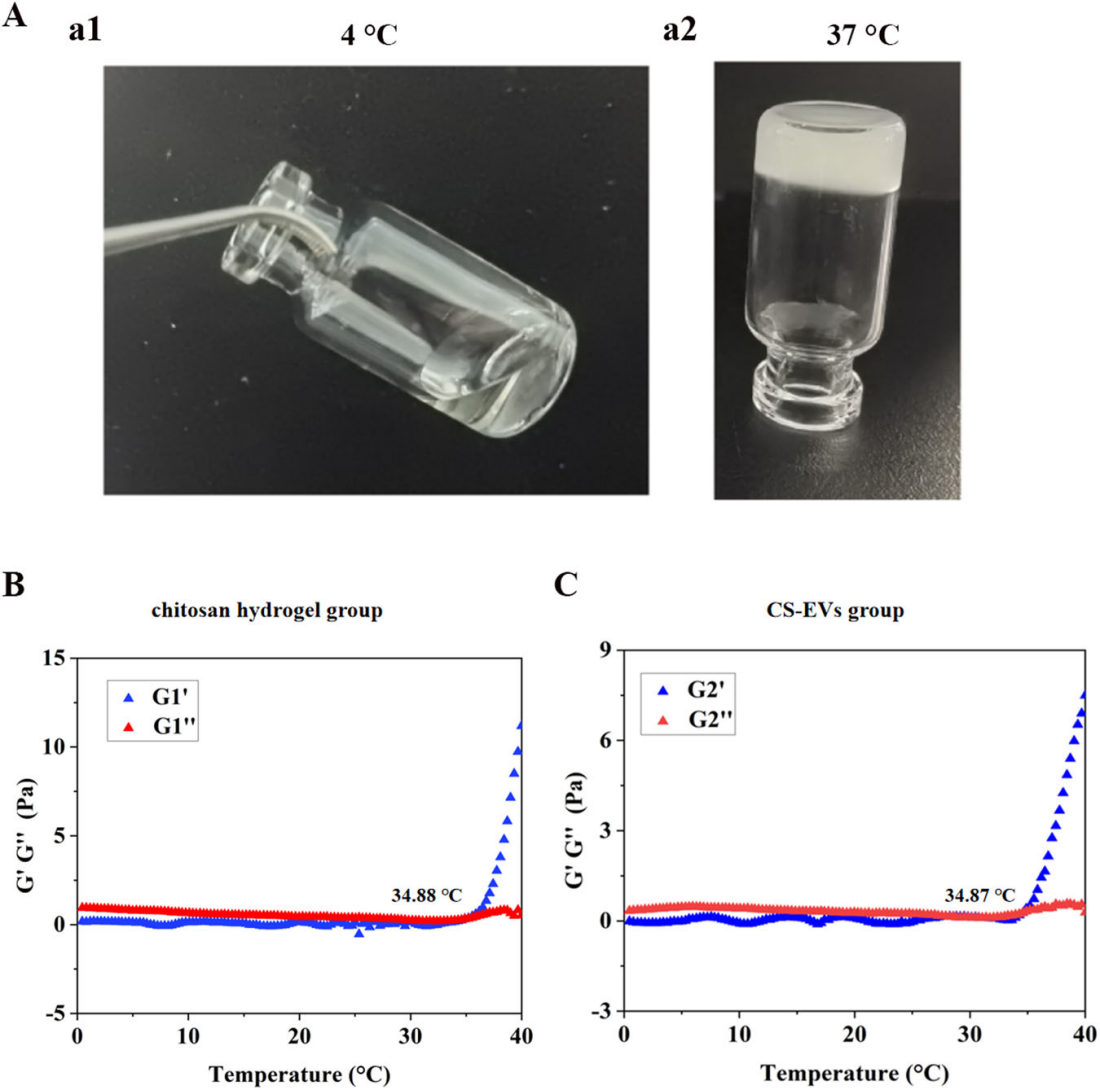
Characterization of chitosan hydrogel and CS-EVs: **A** Images of chitosan solution at 4 °C (a1) and hydrogel at 37 °C (a2). **B** Rheological properties of chitosan hydrogel. **C** Rheological properties of CS-EVs

Prior research has highlighted the suitability of thermosensitive chitosan hydrogel as a biocompatible polymer material for delivering cells and drugs [30]. In the experiment, we utilized liquid chitosan hydrogel at 4 °C, which underwent gelation at 37 °C for 10 min (Fig. 3A). To evaluate the rheological properties of the chitosan hydrogel and CS-EVs, we conducted an analysis and observed that the inclusion of EVs did not affect the formation of the chitosan hydrogel (Fig. 3B, C).

### Release and uptake behavior of EVs leaching from chitosan hydrogel

**Fig. 4:**
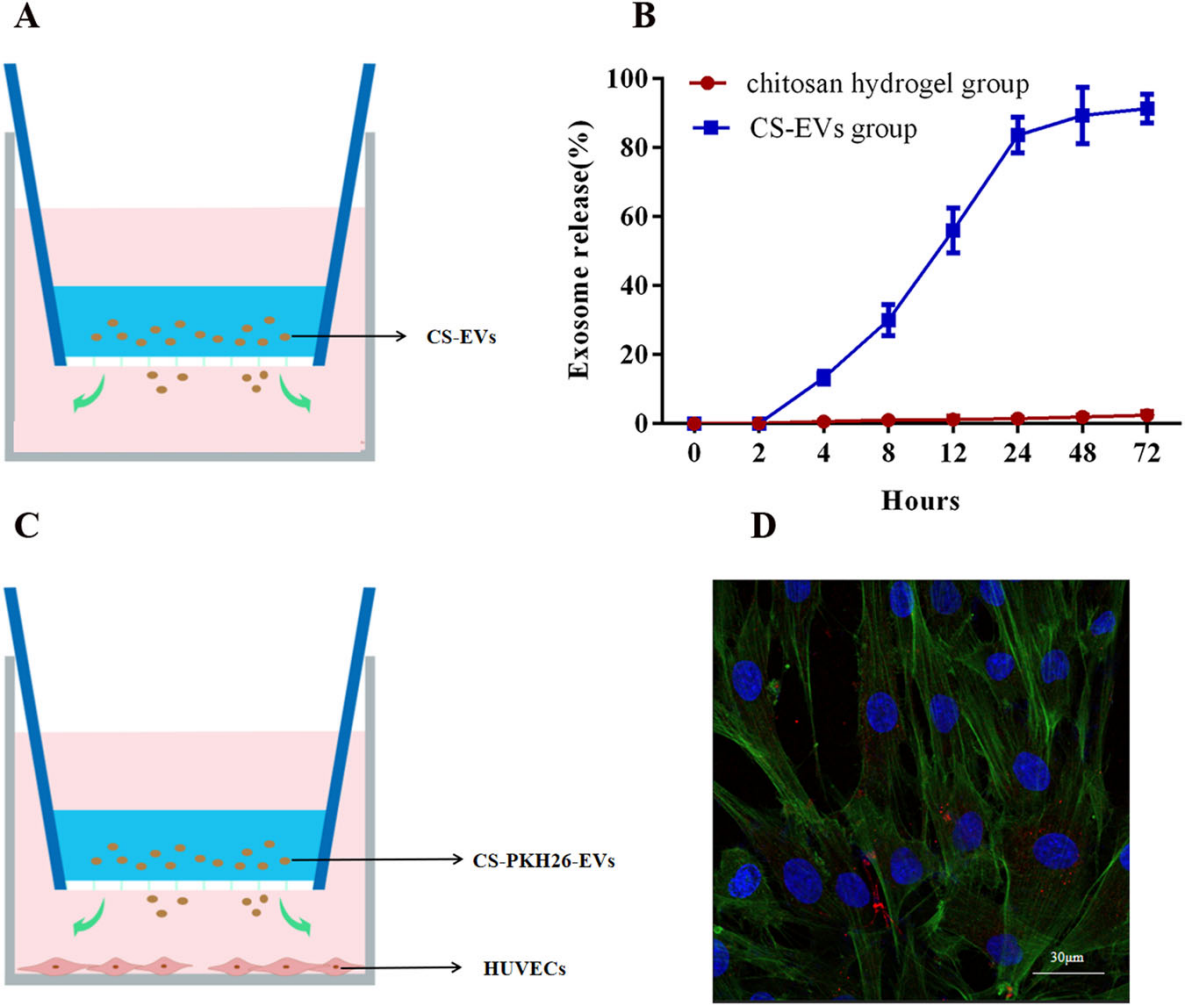
In vitro release and uptake behavior of EVs leaching from the CS-EVs: **A** Schematic representation of CS-EVs in the upper Transwell chamber releasing EVs to the surroundings. **B** The release profiles of EVs leaching from CS-EVs. **C** Schematic representation of CS-PKH26-EVs in the upper Transwell chamber releasing PKH26-EVs to the surroundings. **D** The internalization of PKH26-labeled EVs by HUVECs

Figure 4A illustrates the release pattern of EVs from the hydrogel into the PBS solution. These findings indicate that CS-EVs exhibited sustained release of EVs into the surrounding environment over a period of 72 h. Notably, there was a rapid initial release of EVs from the hydrogel within the first day, with a release rate of 83.67 ± 5.13% over 24 h (Fig. 4B). Furthermore, the EVs leached from the chitosan hydrogel exhibited fusion with HUVEC cells (Fig. 4C, D).

### Proangiogenic capacity of CS-EVs

**Fig. 5:**
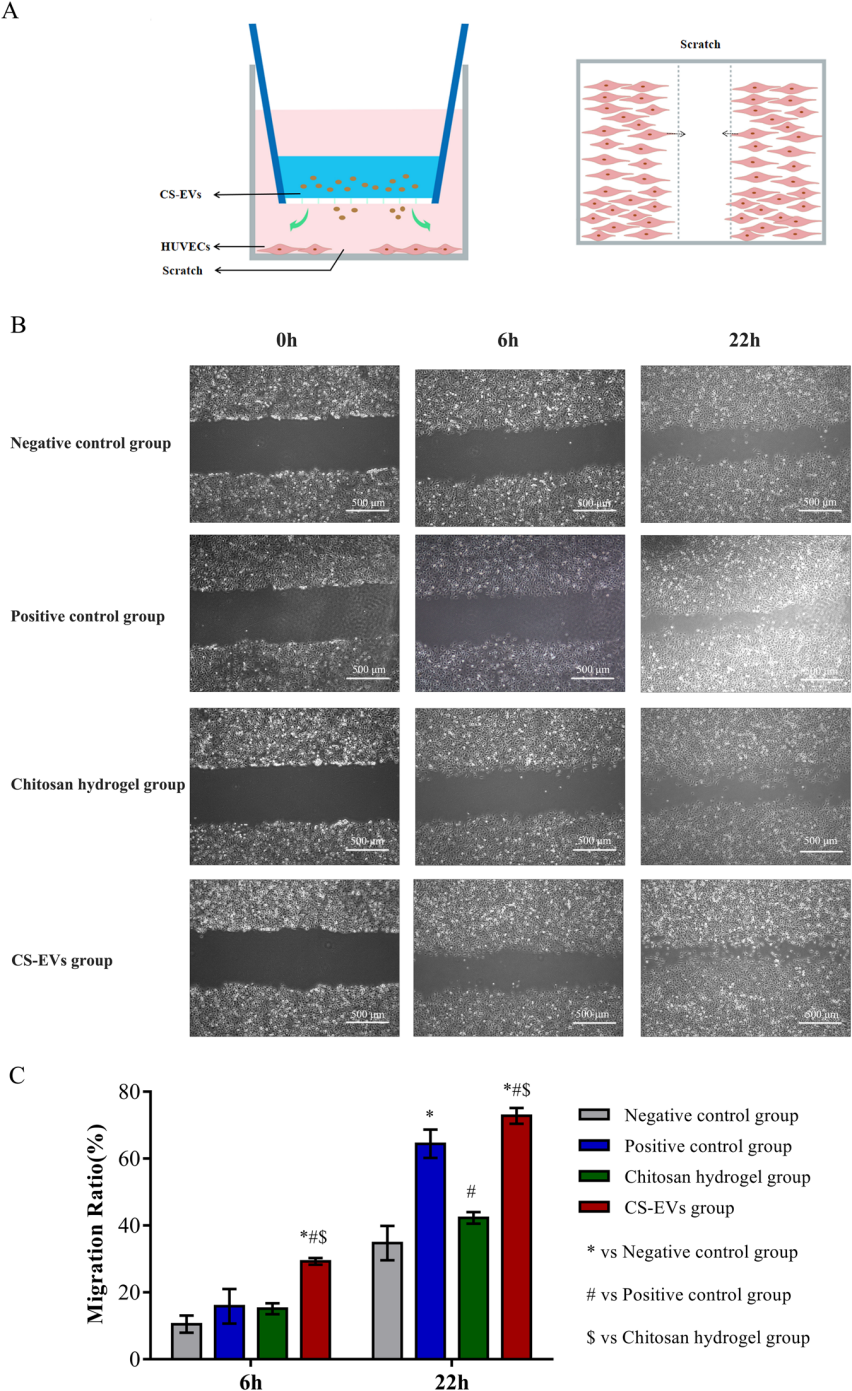
Enhanced migratory ability of CS-EVs in HUVECs: **A** Schematic representation of migration tests. **B** Images showing cell migration in HUVECs at 6 h and 22 h. **C** Quantification of the cell migration rate in HUVECs. All data are presented as mean ± SD; *n* = 6 per time point and group. Statistical analysis using two-way ANOVA revealed: * *p* < 0.05 versus the negative control group; # *p* < 0.05 versus the positive control group; $ *p* < 0.05 versus the chitosan group

**Fig. 6:**
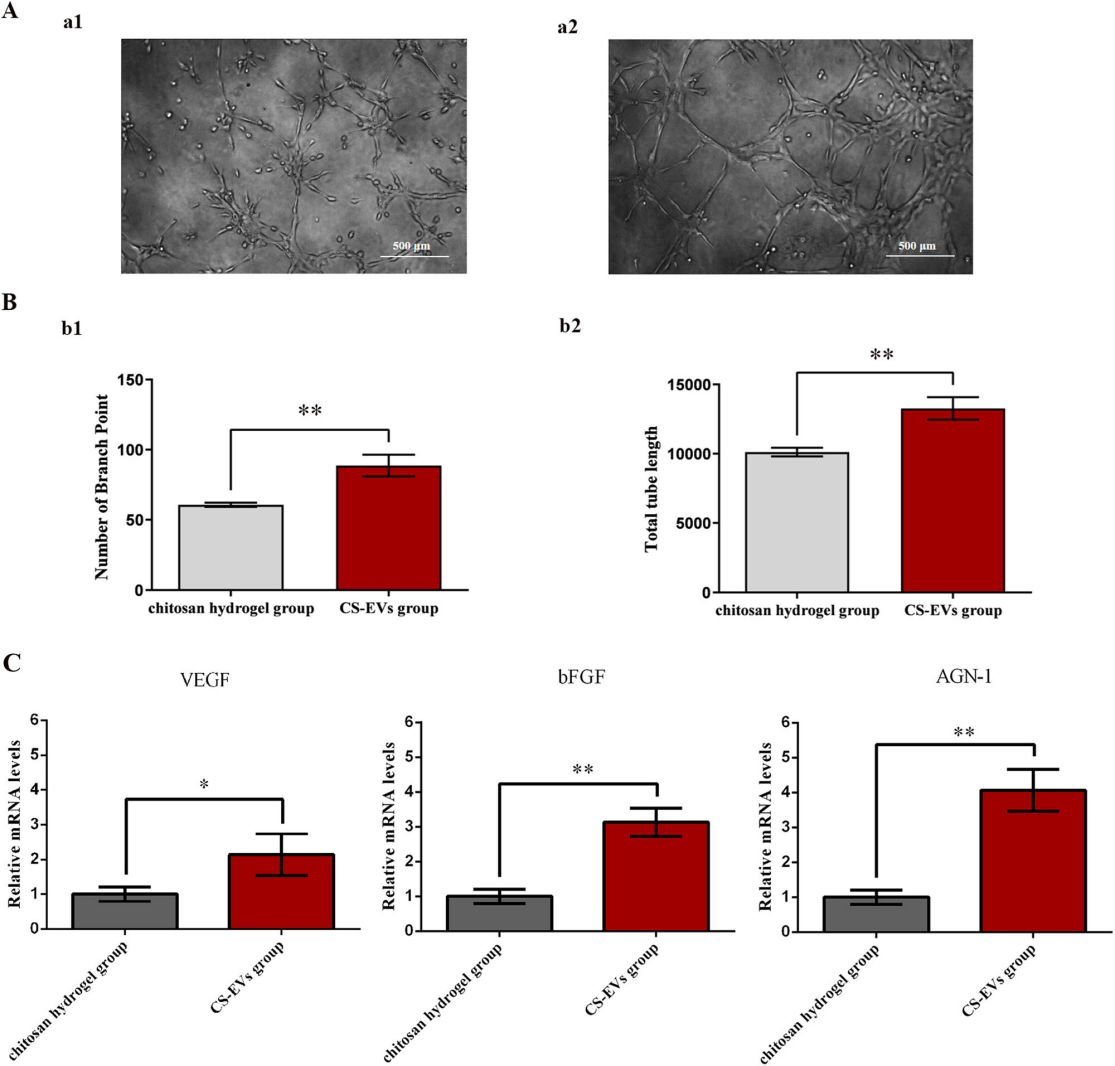
Tube formation assays. **A** CS-EVs enhance the angiogenic ability of HUVECs: a1. Chitosan hydrogel stimulates the tube formation ability of HUVECs; a2. CS-EVs stimulate the tube formation ability of HUVECs. **B** Quantitative analysis of the angiogenic capacity of HUVECs: b1. Branch point number in both groups; b2. Total tube length in both groups. **C** mRNA levels of VEGF, bFGF, and ANG-1. All data are given as mean ± SD; *n* = 6 per time point and group. The asterisks (*) indicate *p* < 0.05, and (**) indicate *p* < 0.01

The in vitro wound-healing assay demonstrated an improved migratory capacity of HUVECs upon the addition of CS-EVs at 6 and 22 h (Fig. 5A, B). Following 22 h of incubation, the wound closure rate in cells treated with CS-EVs (72.8%) was significantly higher compared to those treated with chitosan hydrogel alone (42.3%) (Fig. 5C). Tube formation assays were conducted to assess the proangiogenic potential of CS-EVs, and notable differences in tube length and branch point values were observed compared to the control group (Fig. 6B). Furthermore, the expression levels of VEGF, bFGF, and ANG-1 genes in HUVECs were significantly upregulated (*P* < 0.05) after incubation with CS-EVs (Fig. 6C). These findings indicate that hUCMSC-EVs have the ability to promote angiogenesis by modulating the expression of pro-angiogenic-related genes.

### In vivo evaluation of the closure of a full-thickness wound

**Fig. 7:**
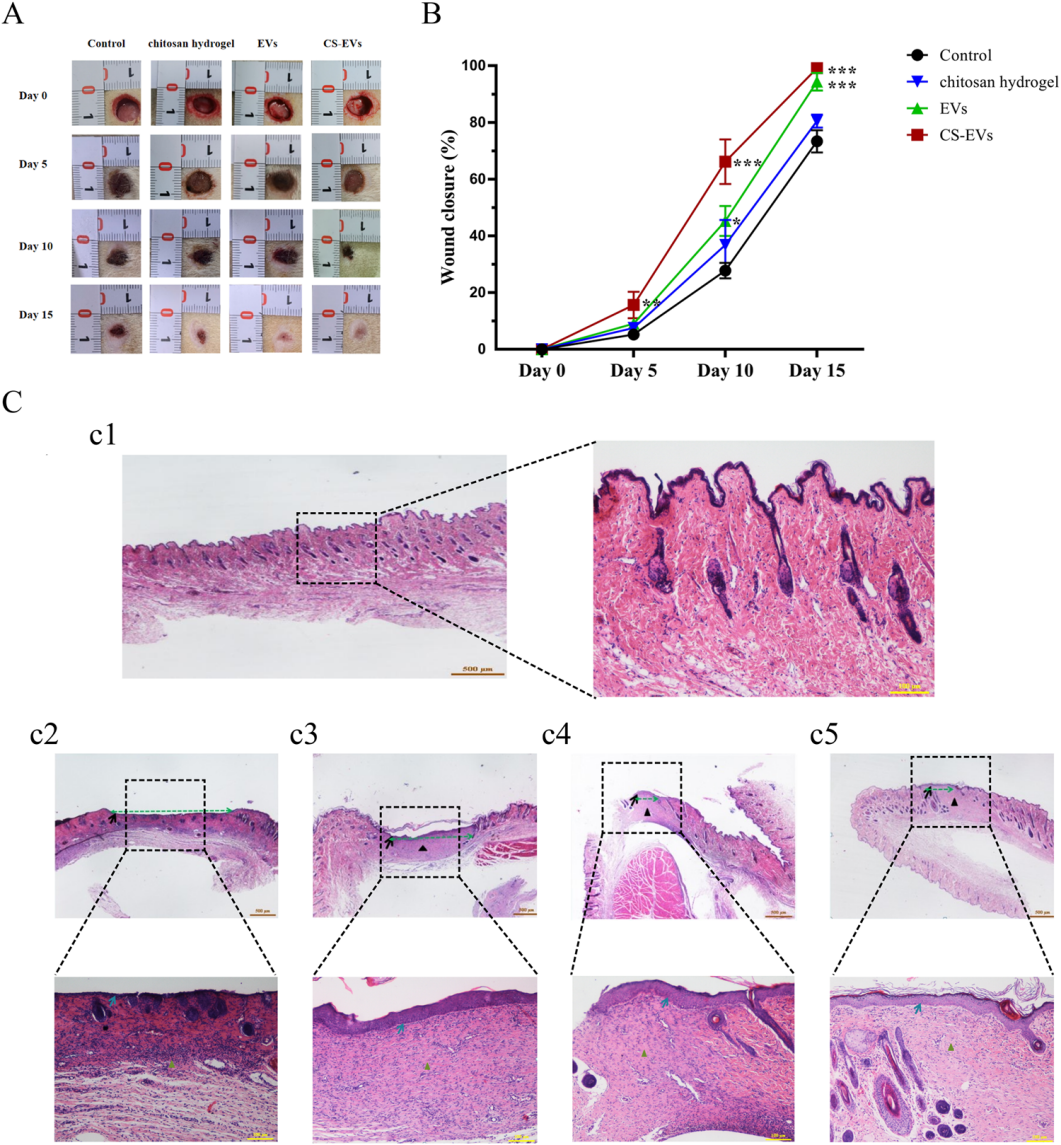
In vivo analysis of wound healing: **A** The wounds were photographed on days 0, 5, 10, and 15. **B** Quantitative analysis of the rates of wound healing. **C** Histopathological analysis of wounds on day 15: c1. Control (normal skin); c2. PBS group; c3. Chitosan hydrogel group; c4. EVs group; c5. CS-EVs group. All data are given as mean ± SD; *n* = 6 per time point and group. The asterisks (*) indicate *p* < 0.05, and (**) indicate *p* < 0.01

To investigate the in vivo proangiogenic and wound healing effects of CS-EVs, we utilized a rat model with full-thickness skin wounds on their backs. Figure 7A displays the wound healing progress on days 0, 5, 10, and 15 for each experimental group. Throughout all time points, the CS-EVs group exhibited the most favorable wound healing outcomes, followed by the EVs group and the chitosan hydrogel group. Conversely, the PBS group showed the poorest healing performance. By day 10, the wounds in each group displayed varying degrees of healing. Following a 15-day treatment period, the CS-EVs group demonstrated an impressive wound closure ability of approximately 93.3%, accompanied by a high degree of re-epithelialization. In contrast, the control group exhibited only a 71.5% reduction in wound size (Fig. 7B). H&E staining on day 15 revealed nearly complete healing in the CS-EVs group, characterized by mature glandular tissue, well-organized collagen fibers, and a dermis resembling normal skin tissue. Notably, CS-EVs significantly facilitated accelerated wound healing through processes such as wound epithelization and tissue remodeling, surpassing the effects observed in the other groups (Fig. 7C).

## Discussion

A diabetic foot ulcer represents a complex and incurable chronic wound that poses a significant threat to both the physical and mental well-being of patients. Diabetic wound healing is characterized by persistent bleeding, disrupted regulation of inflammation, hindered cell proliferation, increased susceptibility to infection, and impaired tissue remodeling [35]. Recently, numerous studies have demonstrated that extracellular vesicles derived from MSC-EVs inherit the potent abilities of their parent cells, including inflammation and immune modulation, promotion of angiogenesis, cell proliferation, and migration, alleviation of oxidative stress, and regulation of collagen remodeling imbalances [36]. MSC-EVs offer the advantage of avoiding potential risks associated with direct stem cell transplantation, making them promising “cell-free” therapies for diabetes and its chronic complications [37–41]. However, the source of EVs, standardized preparation protocols, identification procedures, and determination of purity present potential limitations and clinical challenges. To overcome these hurdles and facilitate the clinical translation of EVs, we opted for human umbilical cord mesenchymal stem cells (hUCMSCs) as the ideal cell source for EV isolation. The methods involved a rapid and cost-effective enrichment of EVs from the cell culture media using polyethylene glycol, followed by ultracentrifugation for further purification. Crucially, we employed various techniques such as TEM, western blotting, and HSFCM analysis to determine the size distribution, particle concentration, and phenotypic characteristics of the extracellular vesicles.

Concurrently, we placed emphasis on ensuring the purity of EV preparations, particularly in the context of therapeutic development. Achieving high purity is a crucial factor in guaranteeing the sensitivity and accuracy of downstream assays. It is worth noting that relying solely on ensemble western blot analysis of CD9, CD63, and CD81 in EV preparations may not be sufficient to claim purity and assess the recovery of EVs. To address this, we employed bivariate fluorescence versus SSC using HSFCM to determine the proportions of EVs positive for CD9, CD63, or CD81. Additionally, we utilized Triton X-100 to disrupt the phospholipid membranes of EVs and quantified the particle counts before and after treatment using HSFCM. The findings indicate that approximately 80% of the isolated EVs obtained from hUCMSC culture supernatants were composed of membrane structures. These results highlight the scientific validity of employing positive labeling and assessing membrane integrity as reliable methods for evaluating EV purity, which can be applied to purity testing of EV products.

Chitosan hydrogel has been recognized as a favorable carrier for EVs due to its ability to create an immune isolation barrier, shielding EVs from clearance by the host immune system [42]. Moreover, chitosan hydrogel exhibits properties that help maintain a moist wound environment and cool the wound surface, making it a promising candidate for wound treatment. Hydrogels serve as modern functional wound dressings, and they exhibit considerable therapeutic effects in diabetic wounds. However, their function in wound treatment is limited to providing a moist, antibacterial healing environment. To enhance wound repair, researchers have explored the synergistic potential of combining EVs with biomaterials to create advanced wound dressings [43]. In the study, we crosslinked EVs with chitosan hydrogel to enhance the therapeutic efficacy of EVs by protecting them from clearance and degradation at the wound site. We successfully prepared CS-EVs, which demonstrated sustained release of EVs into the surrounding environment. To evaluate the impact of chitosan materials on the biological activity of EVs, we conducted a Transwell experiment to confirm that HUVEC cells internalized chitosan released from the chitosan hydrogel. This internalization further promoted the migration and angiogenesis of HUVEC cells. Notably, in animal experiments, the combination of EVs and chitosan hydrogel exhibited superior outcomes compared to the use of EVs alone, effectively promoting skin wound healing in an STZ-induced diabetic rat model.

These findings provide novel insights into the role of hUCMSC-derived EVs in wound healing and introduce a novel, non-invasive application method for EVs that holds practical significance in skin repair. However, it is imperative to address an additional aspect of EV-based biomaterial research, which involves elucidating the underlying mechanisms of action. Therefore, further investigations are necessary to overcome regulatory obstacles and provide a clear understanding of the mechanisms involved before clinical implementation can be achieved. Another limitation of this study is the absence of a functional component analysis of EVs and an exploration of their mechanism in wound repair. Consequently, the focus of future endeavors is to elucidating the precise mechanism by which hUCMSC-derived EVs enhance skin regeneration.

## Conclusions

In conclusion, this study successfully developed a safe and efficient method for the large-scale production of EVs derived from human umbilical cord mesenchymal stem cells (hUCMSC-EVs). The majority of the EVs obtained in the presented protocol displayed either a cup-shaped or round-shaped morphology, with a median diameter of ~73.25 nm. The percentages of CD9-positive, CD63-positive, and CD81-positive EVs were found to be 37.5%, 38.6%, and 19.8%, respectively. By incorporating these EVs into chitosan hydrogel, CS-EVs were developed, which demonstrated sustained release of EVs into the surrounding environment. Importantly, the released EVs from CS-EVs were readily internalized by HUVECs and significantly enhanced their migratory and angiogenic capacities under in vitro conditions. Furthermore, in a rat model of diabetic foot ulcers, CS-EVs demonstrated a remarkable improvement in the rate of wound healing. Following a 15-day treatment period, the CS-EVs group displayed an impressive wound closure ability of nearly 93.3%, accompanied by a high degree of re-epithelialization. In contrast, the control group only exhibited a 71.5% reduction in wound size. Collectively, these findings offer potential solutions for the purification, characterization, and application of EVs in the treatment of wounds.

### Supplementary information

Supplementary Information

## Data Availability

All data generated and analyzed during this study are included in this article.

## Abbreviations

EVs: extracellular vesicles
hUCMSCs: human umbilical cord mesenchymal stem cells
CS-EVs: chitosan hydrogel-incorporated EVs
HUVECs: human umbilical vein endothelial cells
DFU: diabetic foot ulcer
MSCs: mesenchymal stem cells
hEnSCs: human endometrial stem cells
GMSCs: gingival mesenchymal stem cells
DMEM/F12: Dulbecco’s Modified Eagle Medium F-12
FBS: fetal bovine serum
DMEM: Dulbecco’s Modified Eagle Medium
TEM: transmission electron microscopy
*β*-GP: *β*-glycerophosphate
qPCR: quantitative real-time PCR
