# Supplementary figures and images for: Enhanced therapeutic effects of mesenchymal stem cell-derived extracellular vesicles within chitosan hydrogel in the treatment of diabetic foot ulcers
Source: J Mater Sci Mater Med. 2023 Aug 28;34(9):43. doi: 10.1007/s10856-023-06746-y (PMC10462522; doi:10.1007/s10856-023-06746-y)

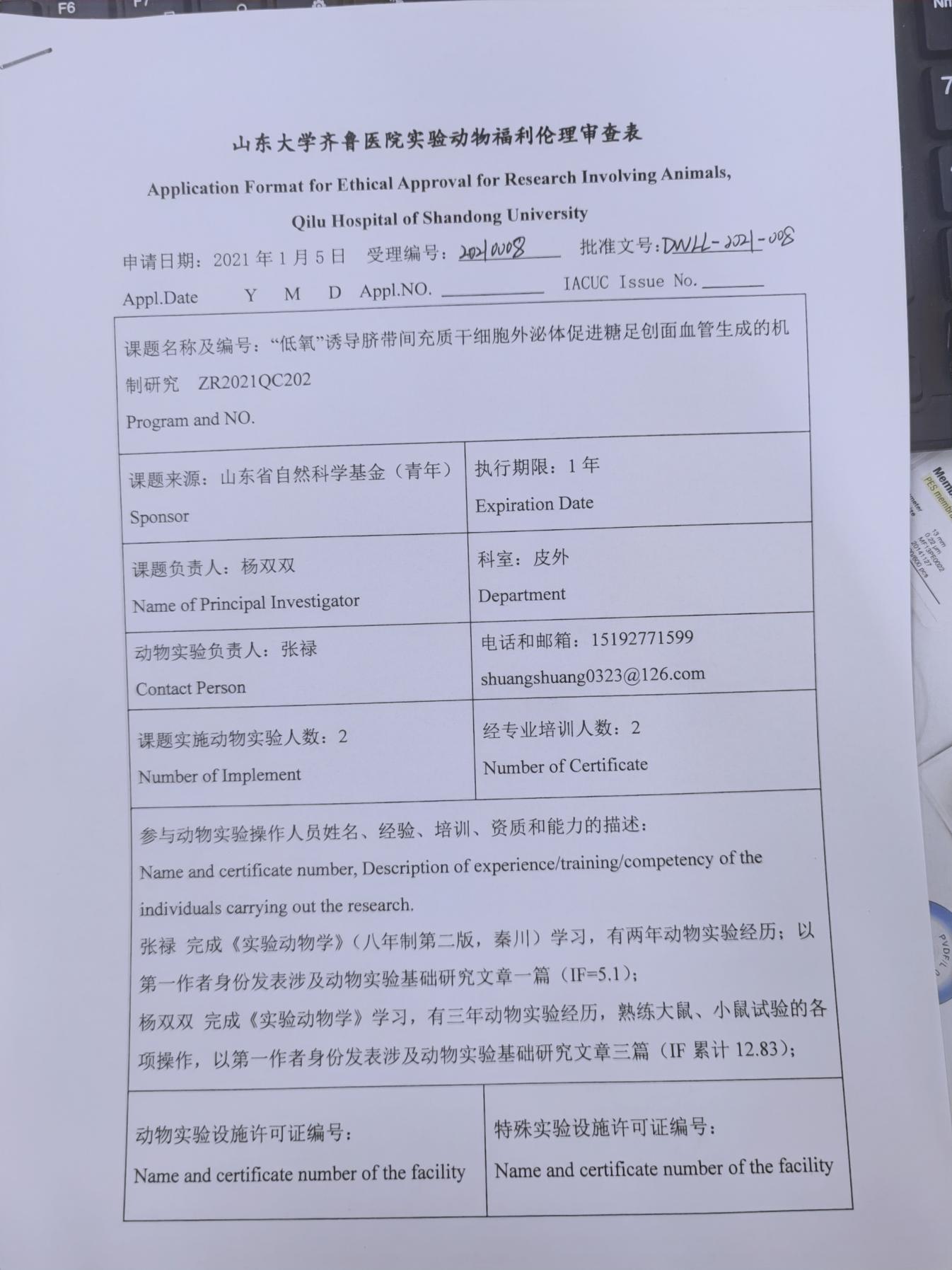


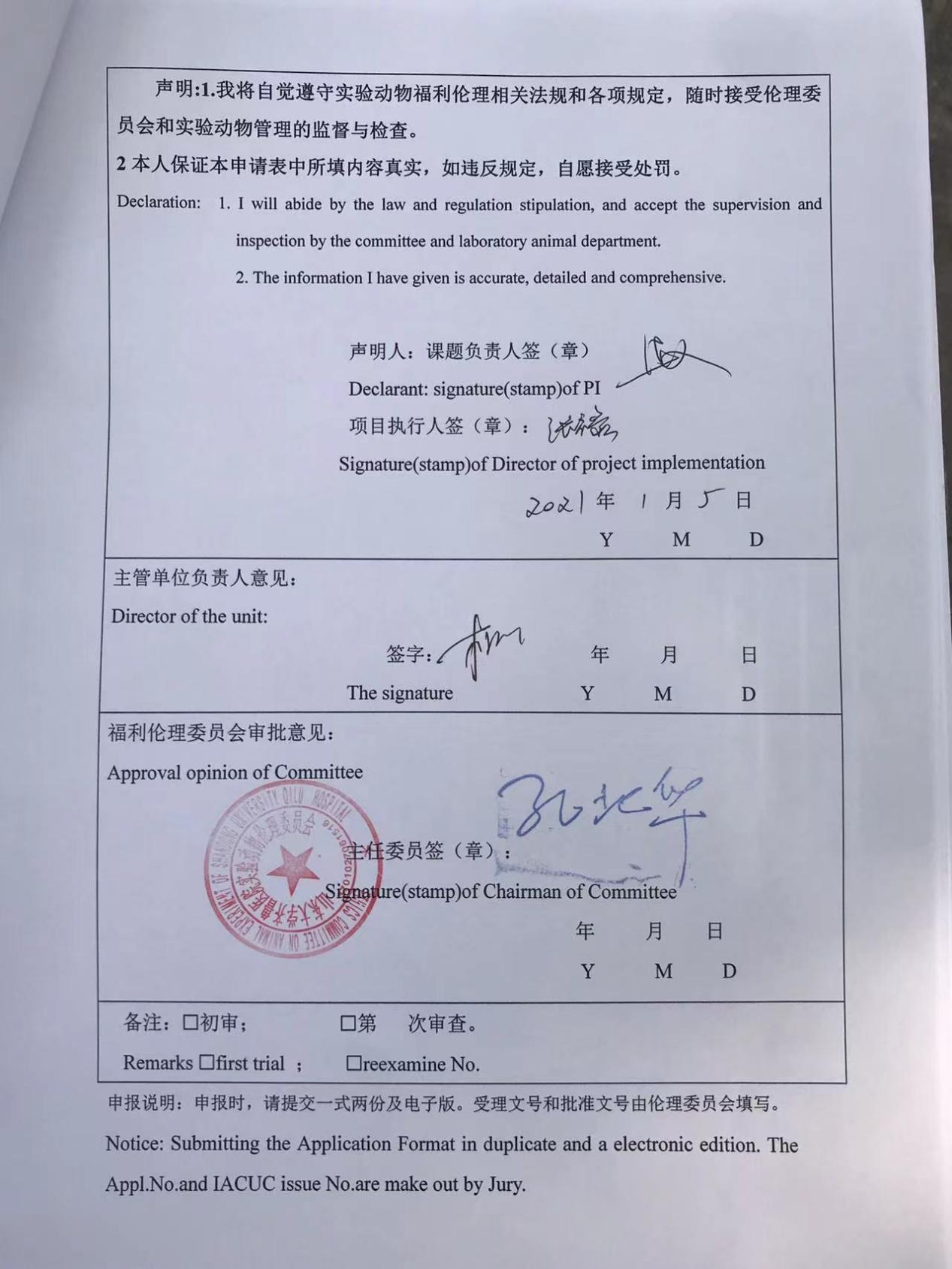

Supplement: Supplementary file 1 — Supplementary Information [file 10856_2023_6746_MOESM1_ESM.docx]
